# Opioid Overdose and Serotonin Syndrome due to Gastric Bezoar in a Woman with Autism and Pica Behaviour

**DOI:** 10.1155/2021/7334467

**Published:** 2021-12-14

**Authors:** Iolanda Palimaru, Michaël Guetta, Cora Cravero, Clémence Fron, David Cohen, Marianna Giannitelli

**Affiliations:** ^1^Department of Child and Adolescent Psychiatry, Reference Centre for Rare Psychiatric Diseases, Pitié-Salpêtrière Hospital, AP-HP.Sorbonne Université, 47-83 Boulevard de l'Hôpital, 75013 Paris, France; ^2^Interdepartmental Mobile Unit for Complex Situations in Autism (UMI 75-92), Elan Retrouvé Foundation, Rue Gager-Gabillot, 75015 Paris, France; ^3^Department of Hepato-Gastroenterology, Pitié-Salpêtrière Hospital, AP-HP.Sorbonne Université, 47-83 Boulevard de l'Hôpital, 75013 Paris, France; ^4^CNRS UMR 7222, Institute for Intelligent Systems and Robotics, Sorbonne University, 4 Place Jussieu, 75005 Paris, France; ^5^Clinical Research Group (GRC) 15, Psychiatric Disorders and Development (PSYDEV), Sorbonne University, 47-83 Boulevard de l'Hôpital, 75013 Paris, France

## Abstract

We are presenting the case of a 38-year-old woman with nonverbal autism and intellectual disability, hospitalized in a neurobehavioural unit because of a pica behaviour for 3 years. During the hospitalization, the patient presented an episode of pain, agitation, restlessness, rhabdomyolysis, coma, tachycardia, hyperthermia, shivering, and diarrhoea. The main hypothesis raised was tramadol overdose because of the immediate antidote response to the injection of naloxone 0,4 mg/mL. Even if we did not exceed the recommended prescription dosage of tramadol, the presence of gastric bezoar slowed the absorption of the drug, and the consequence was an opioid overdose and serotonin syndrome.

## 1. Introduction

Bezoars represent an accumulation of foreign bodies in the gastrointestinal tract. They are most commonly localised in the stomach [1] and could be the consequence of pica behaviour.

Pica can be encountered in several contexts of iron deficiency anemia, pregnancy, psychiatric conditions (e.g., depression, anxiety including trichotillomania, intellectual disability (ID), and autism spectrum disorder (ASD)) and more rarely in postbariatric surgery patients [2, 3]. When pica occurs after the first years of life in a patient with ASD/ID, a somatic etiology should be sought esophagitis and/or gastritis [4]. Bezoars can be asymptomatic, or they can cause nonspecific symptomatology such as nausea, vomiting, abdominal pain, and digestive bleeding [5, 6].

The management of bezoars depends on their size and above all on their impact on patients' well-being. Treatments include the conservative—“watchful waiting”—treatment [7], the dissolution treatment with different compounds such as laxatives or Coke-like soft drinks [8], endoscopic removal, laparotomy, and laparoscopic surgery [8–11].

In patients for whom symptoms suggest a lack of dosage or drug plasma levels exceeding known or expected pharmacokinetics principles, the presence of a foreign mass like a bezoar inside the gastrointestinal tract should be considered. A bezoar could temporarily block the absorption of a drug followed by a subsequent overdose [12].

## 2. Case Presentation

We are presenting the case of a 38-year-old woman with nonverbal autism, epilepsy, and severe ID, who was hospitalized in the neurobehavioural unit of the Pitié-Salpêtrière Hospital because of the installation of a severe pica behaviour for three years. The patient has a history of epilepsy, a treated gastric ulcer, and vitamin B9 and iron deficiency previously supplemented.

**Figure 1:**
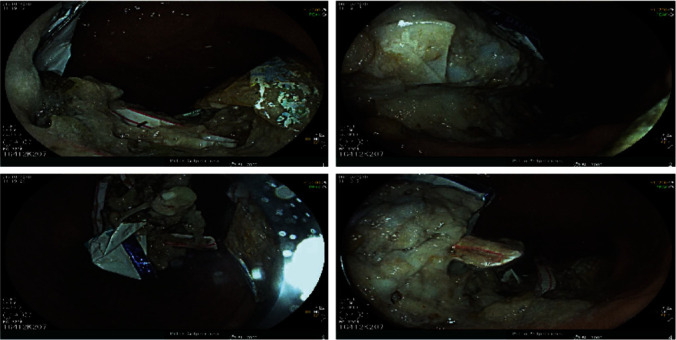
Bezoar pictures of foreign objects during the first gastroduodenal fibroscopy.

After a period of observation in the unit, we realized that the problematic behaviour was the ingestion of inedible foreign objects, which required constant monitoring. In this situation, our patient underwent gastroduodenal fibroscopy under general anaesthesia which showed a gastric bezoar with multiple plastic foreign bodies, especially straws, medical compresses, and gloves (Figure 1). Due to a technical problem, the bezoar could not be completely removed.

At the admission, her current treatment was sodium valproate 1250 mg/d and diazepam 11,66 mg/d (as antiepileptics), sertraline 50 mg/d (as anxiolytic), melatonin 12 mg/d (to improve sleep disorders), sodium alginate/sodium bicarbonate 3 tablets/d, lansoprazole 30 mg/d, and phloroglucinol 480 mg/d (for abdominal pain and gastric protection).

A few weeks later, while we were trying an intensive behavioural therapy (to identify the environmental contingencies that contribute to the maintenance of the problematic behaviour and manipulate these to bring about a desired behaviour change using extinction and positive reinforcement [4], she presented with a deterioration of her general condition. She intensified a compulsive motor activity (repeatedly packing/unpacking her bedclothes), and she became painful on abdominal palpation; in addition, she had diarrhoea. In the hypothesis of a subocclusive syndrome due to her severe pica behaviour, we proceed to a colonic lavage with polyethylene glycol which allowed the elimination of a medical compress, and then, we prescribed tramadol 225 mg/d to calm her pain.

During this episode, the biological assessment showed an inflammatory syndrome (CRP = 62, 32 mg/L (*N* < 5 mg/L)), iron deficiency anemia (hemoglobin = 9, 9 g/dL (*N* = 12-16 g/dL)), disturbance of the hepatic assessment (ASAT = 241 U/L (*N* = 17-27 U/L), ALAT = 93 U/L (*N* = 11-26 U/L)), rhabdomyolysis (CPK = 8046 U/L (*N* < 170 U/L)), and cardiac injury (NT pro-BNP = 846 ng/L (*N* < 300 ng/L), T-troponin = 16, 1 ng/L (*N* < 14 ng/L)). Additionally, the viral PCR was positive for coronavirus OC-43.

One day later, she presented profuse sweating and modified physiological constants: a heart rate at 150 beats/minute, systolic arterial pressure at 130 mmHg, oxygen saturation in ambient air at 91%, and peripheral temperature at 38.2°C. The patient presented several desaturations during the night, and the next morning, we found her in a calm coma, with a Glasgow score of 3. She was transferred to the intensive care unit. The main hypothesis raised was tramadol overdose because of the immediate response to the injection of opioid antidote, naloxone 0,4 mg/mL.

The outcome was favourable, the patient became fully vigilant a few hours after the administration of the antidote, and she was transferred back to our neurodevelopmental unit.

She underwent an exploratory laparotomy two weeks later to completely remove the bezoar. The small intestine was not dilated, and neither stenosis nor bezoar was detected. A gastroscopy under general anaesthesia was performed and found a perfectly free stomach. In the absence of an intra-abdominal foreign body, no additional procedure was performed. We hypothesised that the patient spontaneously eliminated the foreign bodies which could not be removed during the first endoscopy. The staged biopsies of the digestive tract did not show mucosal abnormalities nor the presence of *Helicobacter pylori*.

## 3. Discussion

We contacted the regional pharmacovigilance unit to report this clinical case. In the absence of another clear cause, the chronological, semiological, and bibliographic data do not allow us to rule out the hypothesis of a serotonin syndrome (agitation, restlessness, rhabdomyolysis, coma, tachycardia, hyperthermia, shivering, and diarrhoea) [13]. In this case, the main suspected agent was tramadol. Tramadol overdose in combination with sertraline probably contributed to the apparition of a serotonin syndrome. Even if we did not exceed the recommended prescription dosage of tramadol, the presence of gastric bezoar is assumed to have slowed the absorption of the drug, and the consequence was an opioid overdose.

To our knowledge, we found in the literature only lithium intoxication associated with a trichobezoar [14]. More frequently, drug intoxication is caused by the formation of a pharmacobezoar [12, 15–22].

The treatment of anemia by iron supplementation and intensive behavioural therapy allowed for an improvement in her pica behaviour.

The limit of our clinical interpretation would be the presence in this patient of a bezoar concomitant with the appearance of a viral infection with coronavirus OC-43 and the appearance of a serotonin syndrome, which could represent confounding factors.

The reintroduction of tramadol, in particular in the event of continued sertraline (or another serotonergic antidepressant), should be discussed collegially based on the benefit/risk ratio and existing alternatives and under close surveillance.

## Data Availability

The data used to underpin the findings of this study are included within the paper.
